# Aneurysms of the anterior and posterior cerebral circulation: comparison of the morphometric features

**DOI:** 10.1007/s00701-014-2173-y

**Published:** 2014-07-19

**Authors:** Tomasz Tykocki, Bogusław Kostkiewicz

**Affiliations:** 1 Department of Neurosurgery, Institute of Psychiatry and Neurology, Sobieskiego Street, 02-957 Warsaw, Poland; 2 Department of Neurosurgery, Central Clinical Hospital Ministry of Interior, Warsaw, Poland

**Keywords:** Aneurysms, Morphometry, Anterior, Posterior circulation

## Abstract

**Background:**

Intracranial aneurysms (IAs) located in the posterior circulation are considered to have higher annual bleed rates than those in the anterior circulation. The aim of the study was to compare the morphometric factors differentiating between IAs located in the anterior and posterior cerebral circulation.

**Methods:**

A total number of 254 IAs diagnosed between 2009 and 2012 were retrospectively analyzed. All patients qualified for diagnostic, three-dimensional rotational angiography. IAs were assigned to either the anterior or posterior cerebral circulation subsets for the analysis. Means were compared with a t-test. The univariate and stepwise logistic regression analyses were used to determine the predictors of morphometric differences between the groups. For the defined predictors, ROC (receiver-operating characteristic) curves and interactive dot diagrams were calculated with the cutoff values of the morphometric factors.

**Results:**

The number of anterior cerebral circulation IAs was 179 (70.5 %); 141 (55.5 %) aneurysms were ruptured. Significant differences between anterior and posterior circulation IAs were found for: the parent artery size (5.08 ± 1.8 mm vs. 3.95 ± 1.5 mm; p < 0.05), size ratio (2.22 ± 0.9 vs. 3.19 ± 1.8; p < 0.045) and aspect ratio (AR) (1.91 ± 0.8 vs. 2.75 ± 1.8; p = 0.02). Predicting factors differentiating anterior and posterior circulation IAs were: the AR (OR = 2.20; 95 % CI 1.80–270; Is 270 correct or should it be 2.70 and parent artery size (OR = 0.44; 95 % CI 0.38–0.54). The cutoff point in the ROC curve was 2.185 for the AR and 4.89 mm for parent artery size.

**Conclusions:**

Aspect ratio and parent artery size were found to be predictive morphometric factors in differentiating between anterior and posterior cerebral IAs.

## Introduction

Intracranial aneurysms (IAs) located in the posterior circulation may constitute up to 30 % of all aneurysms [13, 18, 30]. Neurosurgical approaches to the treatment of posterior circulation aneurysms are complicated by their anatomy [25]. The mortality and morbidity after surgical treatment of unruptured posterior circulation aneurysms were estimated as 3.0 % and 12.9 %, respectively [18]. Aneurysms located in the posterior circulation have a higher annual bleed rate than those in the anterior circulation. Data also show that the aneurysm dome size and rate of giant IAs are higher in the posterior circulation [18]. A trend of higher growth rates was found in the posterior circulation, especially among multiple aneurysms [4]. Conditions that affect the differences in the clinical course of IAs depending upon the location have not been definitively revealed. Hemodynamic properties in the aneurysmal complex might be different according to the aneurysm location. It was found that basilar artery aneurysms have the lowest average flow rate comparing to anterior location; as a result, low wall shear stress in the aneurysm dome may relate to the risk of rupture and aneurysm growth [3].

Morphological variations between ruptured and unruptured IAs have been postulated to be predictive in the estimation of the aneurysm risk rupture. A wide range of morphological parameters, such as the size ratio (SR), inflow angle and parent artery geometry, is believed to contribute significantly to determining the risk of aneurysm rupture [6, 20, 26].

Therefore, the authors decided to evaluate different morphometric factors of anterior and posterior cerebral circulation IAs and to investigate whether there are any differences between these two groups of IAs.

## Methods

**Fig. 1:**
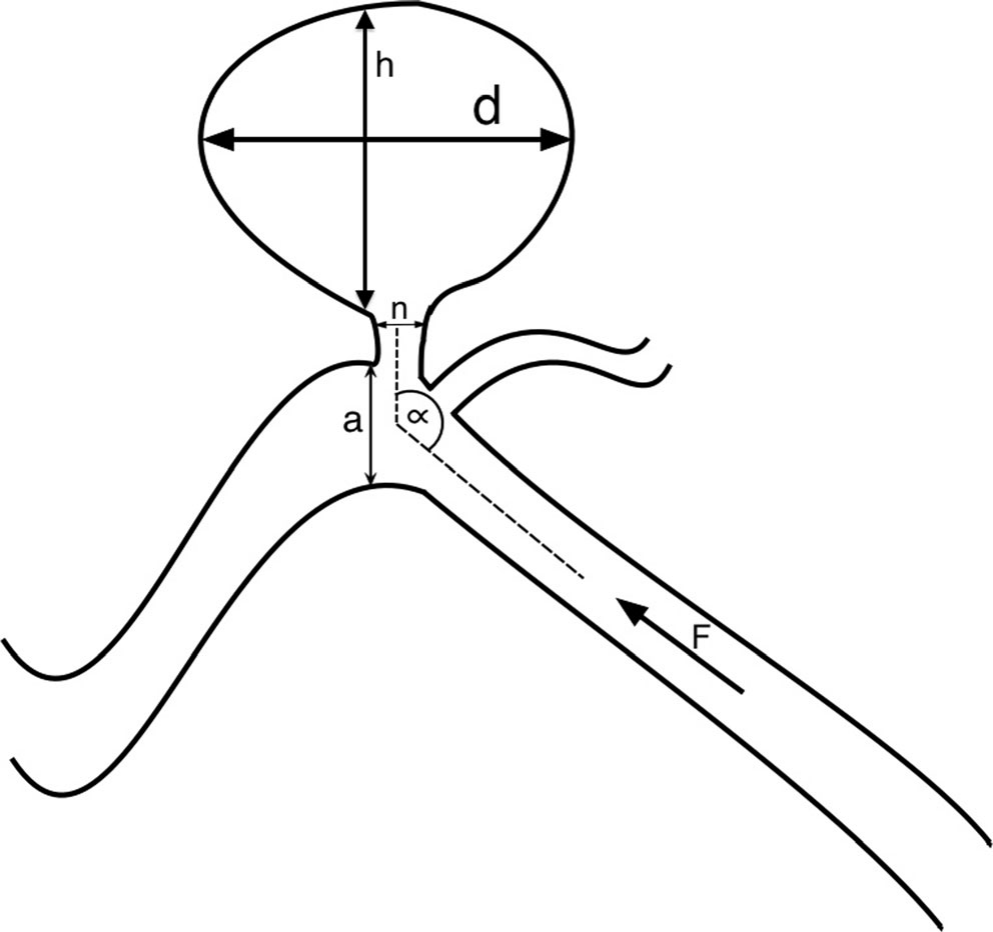
Schematic drawing presenting the morphometric parameters: d, maximal aneurysm dome size; h, aneurysm dome depth; n, neck size; a, parent artery diameter; α, inflow angle; F, blood flow direction; size ratio, d/n; aspect ratio, aneurysm dome depth/neck width

A total of 212 patients (128 females and 84 males) diagnosed with IAs between 2009 and 2012 were incorporated into the retrospective analysis. The patients’ neurological status, demographic and medical history data were based on the retrospective analysis of the medical records. All patients incorporated into the study or their key relatives gave their informed written consent. The following measurements (independent variables) were performed: aneurysm dome size, neck size, parent artery size, size ratio (SR = maximum aneurysm dome size, defined as the largest distance between any two points on the aneurysm dome surface/vessel diameter), neck to parent artery ratio, inflow angle and aspect ratio (AR = aneurysm dome depth/neck width) (Fig. 1). In the aneurysm involving more than one parent vessel, the mean diameter of all vessels was included. IAs were divided according to the location into either the anterior or posterior cerebral circulation. Posterior communicating artery aneurysms originating at internal carotid artery were allocated to the anterior circulation.

## Patient’s selection

All patients were qualified for diagnostic 3D rotational angiography. Three-dimensional reconstruction was performed using an open-source, cross-platform software system for 3D computer graphics (Visualization Toolkit libraries; http://www.vtk.org/). All angiographies within the SAH group were obtained emergently within 48 h of admission to the hospital and prior to vasospasm onset. Size measurements were performed by two neurosurgeons in a blinded fashion with respect to the rupture status of the aneurysms. The intraclass correlation coefficient for two raters was 0.90 (kappa value).

### Statistical analysis

All the results were statistically analyzed using the computer software MedCalc 12.7; Ostend, Belgium. Statistical significance was assumed for P < 0.05. The analysis was performed retrospectively; IAs were assigned to either the anterior or posterior cerebral circulation subsets and incorporated into the statistical analysis.

A t-test was performed to establish the significant differences for independent variables between the anterior and posterior groups. The equality of variances inside the anterior and posterior groups was verified with Levene’s test.

The univariate regression analysis was used for all the independent variables. Finally, the stepwise logistic regression analysis was applied to determine the predictors of the morphometric differences between posterior and anterior circulation IAs (dependent variables). For the defined predictors, ROC (receiver-operating characteristic) curves and interactive dot diagrams were calculated with the cutoff value of the morphometric factor.

Furthermore, the analysis took into account comparison of all ruptured IAs against the location criterion (posterior and anterior circulation). Analogously, the same comparison was conducted for unruptured IAs. For these statistical predictive analyses, multivariate stepwise logistic regression models were used. Interactive dot diagrams were created to establish the cutoff values for the defined morphometric predictors.

## Results

**Fig. 2:**
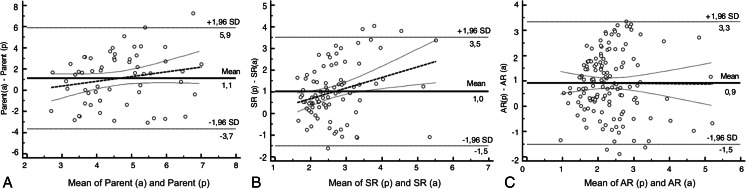
The Bland-Altman plots display a scatter diagram comparing the difference of means of parent artery size (**a**), size ratio (**b**) and aspect ratio (**c**) against their averages in anterior and posterior circulation aneurysms. Horizontal solid lines are drawn at the mean difference, and thin dashed lines are at the limits of agreement, which are defined as the mean difference ± 1.96 times the standard deviation of the differences. The thick dashed line shows the regression curve with the 95 % confidence interval. AR, aspect ratio; SR, size ratio; a, anterior circulation aneurysms; p, posterior circulation aneurysms

**Fig. 3:**
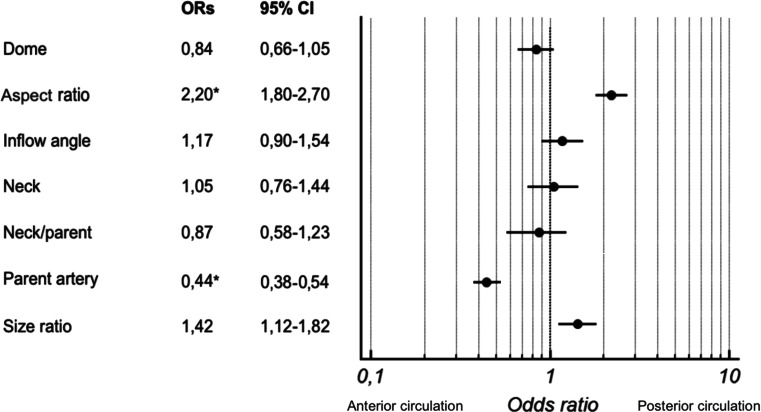
Odds ratios and forest plots for the morphometric factor in the anterior and posterior circulation aneurysms. ORs, odds ratios. ^*^Difference is statistically significant

**Fig. 4:**
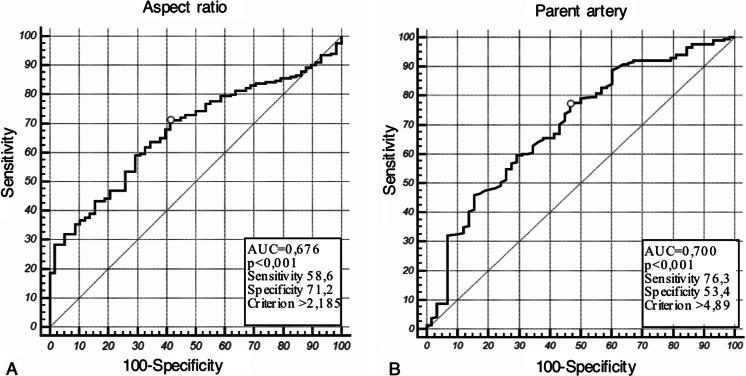
Receiver-operating characteristic (ROC) curves of the aspect ratio (**a**) and parent artery size (**b**) for anterior and posterior circulation aneurysms. Marker indicates the optimal cutoff point differentiating between the two groups

A total of 254 IAs were retrospectively analyzed between 2009–2012; 70.5 % (179) of all IAs were located in the anterior cerebral circulation. The number of ruptured aneurysms was 141 (55.5 %). Levene's test for equality of morphometric factors inside anterior and posterior circulation IAs showed no statistical intergroup heterogeneity (p > 0.05). The mean age of patients was 53.4 ± 12.3 and the male-to-female ratio 0.64. Summary statistics of the morphometric factors of all IAs are presented in Table 1. The comparison of means between the anterior and posterior circulation IAs showed significant differences for three factors: the parent artery size (5.08 ± 1.8 mm vs. 3.95 ± 1.5 mm; p < 0.05) (Fig.2a), SR (2.22 ± 0.9 vs. 3.19 ± 1.8; P < 0.045) (Fig. 2b) and AR (1.91 ± 0.8 vs. 2.75 ± 1.8; p = 0.02) (Fig. 2c) (Table 1). Based on the multivariate stepwise regression model, predicting factors differentiating anterior and posterior IAs were defined. IAs in the posterior circulation were more likely to have over two times higher ARs (OR = 2.20; 95 % CI 1.80–270 Is 270 correct or should it be 2.70 and had over two times smaller parent artery size (OR = 0.44; 95 % CI 0.38-0.54) (Fig. 3) in comparison to those from the anterior circulation. The cutoff point in the ROC curve was 2.185 (sensitivity 58.6 %; specificity 71.2 %) for the AR (Fig. 4a) and 4.89 mm (sensitivity 76.3 %; specificity 53.4 %) for parent artery size (Fig. 4b).

**Table 1:** Morphometric characteristics of all aneurysms and comparison of means of anterior and posterior circualtion aneurysms

	All aneurysms			Anterior circulation aneurysms			Posterior circulation aneurysms			Anterior vs. posterior
	N	Mean	SD	N	Mean	SD	N	Mean	SD	p value
Dome size (mm)	254	12.0	6.9	179	11.13	7.5	75	10.51	3.7	0.292
Aspect ratio	254	2.1	1.2	179	1.91	0.8	75	2.75	1.8	0.020^a^
Inflow angle (degrees)	254	111.2	31.1	179	121.7	22.1	75	109.93	34.8	0.255
Neck size (mm)	254	3.62	4.8	179	3.59	5.3	75	3.68	2	0.485
Neck/parent artery	254	1.2	0.8	179	0.91	0.8	75	1.19	0.2	0.920
Parent artery size (mm)	254	4.2	1.6	179	5.08	1.8	75	3.95	1.5	<0.05^a^
Size ratio	254	3.0	1.0	179	2.22	0.9	75	3.19	1.8	0.045^a^

SD, standard deviation

^a^Difference is statistically significant

**Fig. 5:**
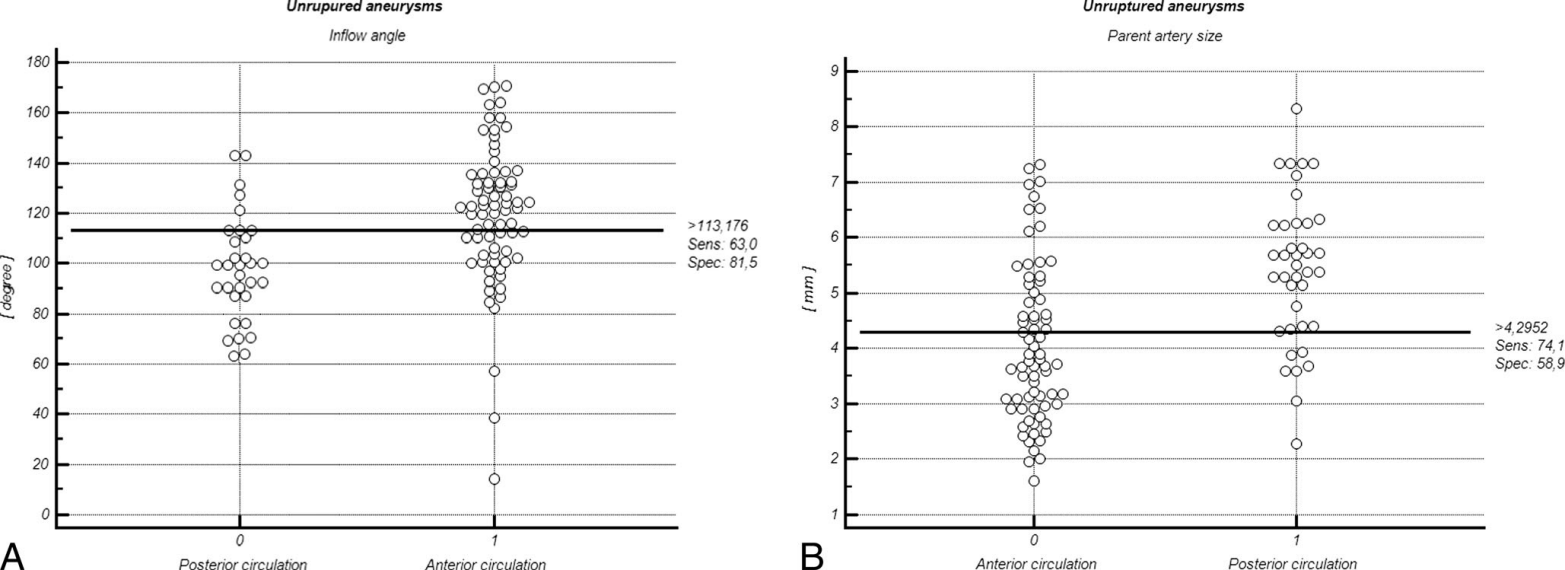
Interactive dot diagram of inflow angle (**a**) and parent artery size (**b**) for the anterior and posterior circulation groups in the population of unruptured aneurysms. In the graph the data of the negative and positive groups are displayed as dots on two vertical axes. Initially, a horizontal line indicates the cutoff point with the best separation (minimal false-negative and false-positive results) between the two groups. The corresponding test characteristics sensitivity and specificity are shown at the right side of the screen display

In addition, the comparison of means in unruptured IAs group showed a significantly larger inflow angle by 12.4 % (119.8 ± 18.3 vs. 106.6 ± 30.6°; p = 0.045) and parent artery size by 35.9 % (5.3 ± 1.4 vs. 3.9 ± 1.7 mm; p = 0.014) in anterior circulation IAs, but the SR was higher in posterior circulation IAs (2.9 ± 1.4 vs. 2.0 ± 0.5; p = 0.022) (Table 2). After the multivariate stepwise regression model had been calculated, it was found that IAs located in anterior circulation were 2.12 times more likely to have a higher inflow angle (Fig. 5a) (cutoff: 113.2°; specificity, 81.5 %; sensitivity, 63 %) and 1.92 times more likely to have larger parent artery size (cutoff: 4.3 mm; specificity, 58.9 %; sensitivity, 74.1 %) (Fig. 5b) than in posterior circulation (Table 2).

**Table 2:** Comparison of means of the morphometric features and odds ratios in the population of the ruptured and unruptured aneurysms categorized by the location in the anterior and posterior cerebral circualation

	Unruptured aneurysms								Ruptured aneurysms							
	Anterior circulation N = 82		Posterior circulation N = 31		p	m	m	p	Anterior circulation N = 97		Posterior circulation N = 44		p	ORs	95 % CI	p
	Mean	SD	Mean	SD					Mean	SD	Mean	SD				
Dome size (mm)	10.1	3.1	10.7	5.1	0.558	1.04	0.67−1.61	0.862	13.89	5.36	10.26	4.0	0.048^a^	0.44	2.03-2.44	0.045^a^
Aspect ratio	1.83	0.7	2.8	1.3	0.151	1.57	1.22−1.87	0.125	2.19	0.9	2.73	1.32	0.535	1.39	0.82-1.84	0.657
Inflow angle (degrees)	119.8	18.3	106.6	30.6	0.045^a^	0.47	0.45−0.92	0.015^a^	125.88	20.6	111.85	36.70	0.079	0.81	0.67- 1.01	0.502
Neck size (mm)	3.4	1.5	3.6	2.0	0.914	1.09	0.66- 1.47	0.950	3.77	2.5	3.73	6.35	0.580	1.25	0.71-1.43	0.680
Neck/parent artery	0.8	0.1	1.2	0.4	0.240	1.43	1.13−1.67	0.268	0.96	0.3	1.36	0.97	0.097	1.58	1.36-1.88	0.827
Parent artery size (mm)	5.3	1.4	3.9	1.7	0.014^a^	0.52	0.34−1.27	0.011^a^	5.06	1.52	3.96	1.9	0.020^a^	0.45	0.22-1.16	0.032^a^
Size ratio	2.0	0.5	2.9	1.4	0.022^a^	1.68	1.34−1.97	0.148	2.58	1.88	3.24	0.7	0.098	0.79	0.57-1.12	0.350

SD, standard deviation; ORs, odds ratios

^a^Difference is statistically significant

**Fig. 6:**
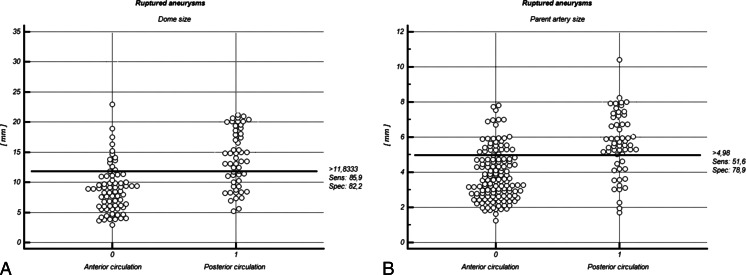
Interactive dot diagram of dome size (**a**) and parent artery size (**b**) for the anterior and posterior circulation groups in the population of ruptured aneurysms. In the graph the data of the negative and positive groups are displayed as dots on two vertical axes. Initially, a horizontal line indicates the cutoff point with the best separation (minimal false-negative and false-positive results) between the two groups. The corresponding test characteristics sensitivity and specificity are shown at the right side of the screen display

Comparison of morphometric factors in ruptured IAs revealed significantly larger dome size (13.89 ± 5.36 vs. 10.26 ± 4.0 mm; p = 0.048) and parent artery size (5.06 ± 1.52 vs. 3.96 ± 1.9; p = 0.020) in anterior circulation IAs (Table 2). Based on the logistic regression analysis, the likelihood of larger dome size among anterior circulation IAs was over two times that in the posterior circulation (cutoff value: 11.8 mm; specificity, 82.2 %; sensitivity, 85.9 %) (Fig. 6a). The probability of larger parent artery size in anterior location was 2.2 times that in the posterior location (cutoff value: 5.0 mm; specificity, 51.6 %; sensitivity, 78.9 %) (Fig. 6b) (Table 2).

## Discussion

The population of IAs varies in the clinical course when taking into account the location of aneurysms, whether in the anterior or posterior cerebral circulation. Generally, well-known results show that the posterior circulation IAs are characterized by an increased risk of bleeding and larger dome size [1, 30]. In light of these facts, an intriguing issue is an attempt to explain the reasons for these differences. Although many studies in the literature are consistent regarding the differences between the aneurysms of different locations, there is no evidence or plausible hypothesis to explain the variabilities. In this study, the authors decided to analyze the morphometric factors of IAs in the anterior or posterior circulation. The authors hypothesized that morphometric features might be potentially differentiating in the properties of these groups and could explain their distinct hemodynamic characteristics.

Based on the results of this study, the location of IAs in the anterior or posterior circulation is associated with distinct morphometric features. The AR and parent artery size turned out to be particularly predictive and were over two times higher among the posterior circulation IAs. The cross comparison of unruptured IAs from both locations showed that the inflow angle is much higher in the anterior circulation, but parent artery size is larger in the posterior group. Similar analysis for ruptured IAs alone also confirmed the larger size of the parent artery in posterior circulation IAs, but additionally indicated a larger dome size in this group. Summarizing the above-mentioned data, parent artery size plays a major role in differentiating anterior and posterior circulation IAs. Other morphometric factors such as AR, inflow angle and dome size are also predictive, but less convincingly.

Morphometric parameters of the aneurysmal complex have been repeatedly investigated mainly in the context of the risk of aneurysm rupture. However, obtaining any data from the literature, comparing these parameters between the anterior and posterior circulation IAs, is challenging. Comparison of ARs from different locations showed that IAs in the anterior circulation had a mean AR of 2, and the mixed group, including the anterior and posterior circulation IAs, had 2.25. The difference in the AR was more pronounced in ruptured aneurysms (2.5 anterior vs. 3.1 posterior circulation) [29]. Discrepancies among other morphometric factors between the anterior and posterior circulation have been proven. Parent artery size was found to be larger in the anterior circulation IAs (3.16 vs. 2.44). In the same study the authors reported an SR of 2.91 in the anterior and 3.56 in the posterior circulation [11]. On the other hand, studies analyzing anterior and posterior IAs independently demonstrated the opposite results for the SR (3.25 anterior vs. 2.26 posterior circulation) [9, 27]. A similar comparison showed a larger inflow angle in the anterior circulation (130.8 vs. 115.2), but the neck/parent artery ratio was higher in posterior circulation (3.45 vs. 1.3) [14, 27].

Results from this study highlight the morphology of the parent artery as reflecting the differences between the anterior and posterior circulation IAs. As the basilar tip IAs accounted for more than 60 % of all IAs in the posterior circulation, the mean parent artery size was shifted toward the larger size. The mean diameter of the basilar artery at the bifurcation was 5.94 mm (data not shown), whereas for all IAs in this location the value was 5.08 mm. In the anterior location the mean parent artery sizes in the middle, anterior and internal carotid arteries were proportionally smaller (MCA 3.4 mm; ACA = 2.2 mm, AcomA = 1.7; ICA = 4.6 mm). The variations in artery diameter were similar to those described in anatomical studies [7, 10, 15, 16]. In other reports the predictive role of the basilar artery diameter was emphasized in the risk of cerebrovascular mortality. A diameter of 4.3 mm may be a marker for a high risk of fatal stroke [17]. The increase in the diameter of basilar artery increases the risk of cerebrovascular events by 1.55 [24].

Sekhar et al. reported a series of 100 basilar tip aneurysms, finding similar AR values in unruptured (clipping = 1.5; coiling = 1.4) and ruptured (clipping = 1.2, coiling = 1.6) [21]. Ujiie et al. showed that unruptured IAs are more predisposed to have an AR greater than 1.6 [29]. Beck et al. reported the opposite results; unruptured IAs had two times higher ARs than ruptured IAs (mean, 2.3 vs. 1.8) [1]. Parameters of the AR are reflected in the intra-aneurysmal flow pattern. Critically slow-velocity flow conditions are observed in an aneurysmal dome with an aspect ratio of more than 1.6. Ujiie et al. found that significant low-flow regions, a possible cause of atherosclerotic inflammation, develop with increasing ARs [28]. Flow stagnation results in the aggregation of red and white blood cells, adhesion of platelets along the intimma and dysfunction of flow-induced nitric oxide, which is usually released by mechanical stimulation through increased shear stress [5, 23]. This pathological cascade causes intimal damage, thrombus formation and subsequent thrombolysis with inflammatory changes in the aneurysm wall [22]. A degenerative, thin structure of the aneurysm wall is less susceptible to contraction in reaction to physiological tensile. A noun is missing after "tensile" and is more prone to rupture.

The retrospective nature of this study is not powered enough to estimate the risk of aneurysm rupture, mostly due to the post hoc morphometric analysis of the ruptured IAs. Some authors report that unruptured aneurysms might not shrink when they rupture [19]. According to others, IAs might reduce their size after rupture [12], but it is conceivable that the shape might change. However, data from this study undoubtedly demonstrate significant morphometric differences between ruptured and unruptured IAs and allow putting forward the hypothesis that they may predict the risk of aneurysm rupture. Tremmel et al. also reported the predictive value of the SR with a cutoff point of 2.0 [26]. In another study, the SR was 2.76 and 69 % of ruptured IAs had an SR > 3 [20]. Xiang et al. showed that for a unit increase in the SR of IAs, the odds of IA rupture increased by 2.96 times [31].

However, there have been discrepancies in determining the optimal value of the AR beyond which aneurysms are deemed to have a high risk of rupture. The AR may range from 1.18 up to 2.7 [6, 18, 29], showing ambiguity and a large scattering of results, thereby diminishing the rank, strength and power of this index. Hence, the AR cannot be taken as a standalone method for risk stratification. Carter et al. hypothesized that the size of the aneurysm may be related to Laplace’s law, which states that the “critical” size for aneurysm rupture is related to the parent artery wall thickness [2]. Therefore, size alone is insufficient to accurately predict the IA rupture risk, and the vessel diameter must also be considered.

The SR is correlated with a variant intra-aneurysmal hemodynamic pattern. Based on the computational fluid dynamics (CFD) studies of intra-aneurysmal hemodynamics, in IAs with an SR above 2, the simple, stable vortex patterns become complex with multiple vortices with higher exposure to low wall shear stress (WSS) [26]. The SR was also inversely correlated with a low WSS [31] defined as the areas of the aneurysm wall exposed to a WSS below 10 % of the mean parent arterial WSS and then normalized by the dome area [8].

Different morphometric predictors have been discussed in the literature; some of them, such as the SR, AR or inflow angle, have been consistently proven to be significant predictors in subsequent studies. However, in several reports conclusions about the prediction of the aneurysm rupture are based on retrospective analysis, which obviously has no power to define the risk of rupture. Nevertheless, considering pooled results from the literature, strong evidence could be found supporting the significant role of the morphometric predictors in the prognosis of the aneurysm rupture risk. The transfer of this knowledge to the clinical routine would facilitate decision-making for the treatment of unruptured aneurysms. This may be particularly useful in challenging cases, when consideration of the predictive morphometric features and estimation of the potential rupture risk provide additional data supporting the decision concerning proper aneurysm management. Furthermore, the location of IAs should be scrupulously considered when making treatment decisions.

## Conclusions

The current study has identified significant morphometric predictors differentiating between IAs located in the anterior and posterior cerebral circulation. The aspect ratio was found to be significantly higher in posterior circulation IAs, but the size of the parent artery was over two times larger in anterior circulation IAs. These two morphometric factors are predictive in differentiating aneurysms in the anterior and posterior cerebral circulation. When only unruptured IAs were included, the inflow angle and parent artery size were larger in the anterior circulation. Similar analysis for only ruptured IAs showed larger parent artery and dome size in anterior circulation aneurysms.
